# Gonorrhoea on the rise in Denmark since 2022: distinct clones drive increase in heterosexual individuals

**DOI:** 10.2807/1560-7917.ES.2024.29.7.2400059

**Published:** 2024-02-15

**Authors:** Thomas Roland Pedersen, Maria Wessman, Mikkel Lindegaard, Søren Hallstrøm, Casper Westergaard, Inger Brock, Esad Dzajic, Dennis Back Holmgaard, Christian Salgård Jensen, Ulrik Stenz Justesen, Jette Brommann Kornum, Turid Snekloth Søndergaard, Marianne Kragh Thomsen, Henrik Westh, Claus Østergaard, Steen Hoffmann, Marc Stegger

**Affiliations:** 1 Department of Bacteria, Parasites and Fungi, Statens Serum Institut, Copenhagen, Denmark; 2 Department of Infectious Disease Epidemiology and Prevention, Statens Serum Institut, Copenhagen, Denmark; 3 Department of Clinical Microbiology, Herlev and Gentofte Hospital, Herlev, Denmark; 4 Clinical Diagnostic Department, University Hospital of Southern Denmark, Esbjerg, Denmark; 5 Department of Clinical Microbiology, Zealand University Hospital, Slagelse, Denmark; 6 Department of Clinical Microbiology, Copenhagen University Hospital - Rigshospitalet, Copenhagen, Denmark; 7 Department of Clinical Microbiology, Odense University Hospital, Odense, Denmark; 8 Department of Clinical Microbiology, Aalborg University Hospital, Aalborg, Denmark; 9 Department of Clinical Microbiology, University Hospital of Southern Denmark, Aabenraa, Denmark; 10 Department of Clinical Microbiology, Aarhus University Hospital, Aarhus, Denmark; 11 Department of Clinical Medicine, Aarhus University, Aarhus, Denmark; 12 Department of Clinical Microbiology, Copenhagen University Hospital, Hvidovre, Denmark; 13 Department of Clinical Medicine, University of Copenhagen, Copenhagen, Denmark; 14 Department of Clinical Microbiology, University Hospital of Southern Denmark, Vejle, Denmark; 15 Antimicrobial Resistance and Infectious Diseases Laboratory, Harry Butler Institute, Murdoch University, Perth, Australia

**Keywords:** Neisseria gonorrhoeae, gonorrhoea, women, MSM, heterosexual, genomics

## Abstract

A surge in gonorrhoea in Denmark has occurred since 2022, a 46% increase from 2021. National surveillance, leveraging mandatory reporting and epidemiological data, highlights three distinct clades linked to heterosexual transmission. Despite the rise, these exhibit high susceptibility, contrasting MSM-associated strains. Geographical hotspots and age-specific patterns further illuminate transmission dynamics. The combination of genomic and epidemiological data provides novel insights into the evolving landscape of gonorrhoea, indicating potential shifts in infection dynamics and transmissibility.

*Neisseria gonorrhoeae* is one of the most common pathogens responsible for sexually transmitted infections (STIs) globally, with over 80 million annual cases worldwide [1]. In Denmark, surveillance of gonorrhoea has shown a drastic increase in cases from 2022 onwards, similar to other countries across Europe [2]. In Denmark, this has particularly been observed in the Capital region and in the Northern region of Jutland in women and heterosexual men, i.e. men who have sex with women (MSW). Here, we use national surveillance data and genomics to aid our understanding of dissemination and potential drivers of *N. gonorrhoeae* infections. 

## National surveillance of *Neisseria gonorrhoeae* infections

An increase in *N. gonorrhoeae* infections has recently been reported across the European continent, according to the European Centre for Disease Prevention and Control [3]. The case definition of gonorrhoea is based on positive PCR or culture, limited to one case within 21 days. In Denmark, data from the national microbiology database (Den Danske mikrobiologidatabase, MiBa) [4] depicted a dramatic increase of 46% in gonorrhoea cases from 2021 to 2022. Denmark’s national surveillance of gonorrhoea is based on mandatory reporting by the departments of clinical microbiology (DCMs), which report positive as well as negative laboratory analyses for *N. gonorrhoeae* to MiBa. Additionally, it is mandatory for clinicians to submit an epidemiological notification for each clinical case of gonorrhoea linking the patient’s personal identification number (PIN) [5] to demographic information, i.e. date of birth, place of birth, sex, residence, as well as information about mode of infection, country of infection and treatment. National surveillance data show that 65–70% of cases of gonorrhoea have an epidemiological notification and that the distribution of epidemiological notified cases of gonorrhoeae in Denmark between 2018 and 2023 showed that women accounted for 35% of all cases and had a lower median age (23 years; range: 0–78) compared to infected men (30 years; range 13–82).

Further, the DCMs voluntarily submit *N. gonorrhoeae* isolates to Statens Serum Institut (SSI), which works under the auspices of the Danish Ministry of Health, for national surveillance of antimicrobial susceptibility. Susceptibility testing to ceftriaxone, ciprofloxacin and azithromycin as well as detection of beta-lactamase production is performed at SSI using E-test, cefinase disks and chocolate agar plates according to EUCAST guidelines [6]. The DCMs are also required to report the number of laboratory-confirmed analyses for *Chlamydia trachomatis* infections, a detection that is performed using a duplex PCR that tests for both *C. trachomatis* and *N. gonorrhoeae*.

## Increase in gonorrhoea and chlamydia cases since 2022 

**Figure 1:**
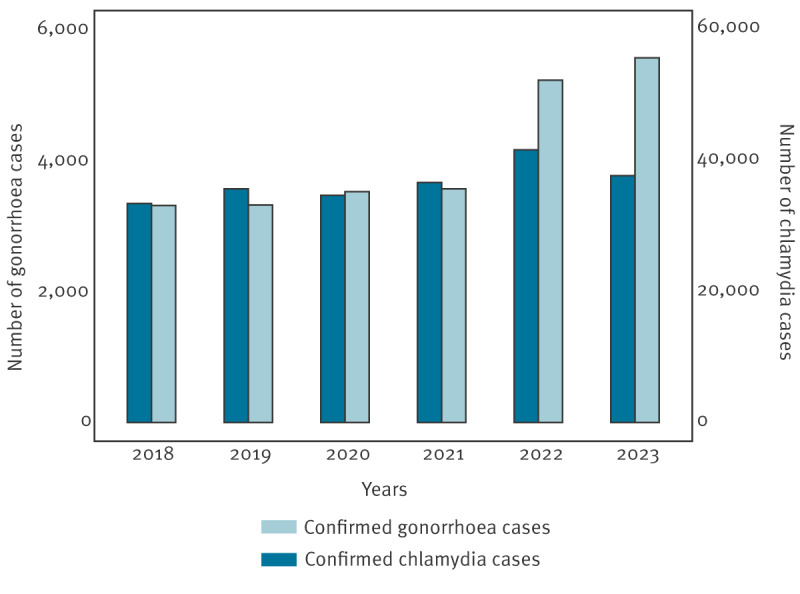
Number of laboratory-confirmed cases of *Neisseria gonorrhoeae* (n = 24,516) and *Chlamydia trachomatis* (n = 219,748), Denmark, 2018–2023

Based on national surveillance data, an increase in the number of cases of both gonorrhoea and chlamydia has been observed during the last 5 years (Figure 1). Whereas the number of chlamydia cases usually is almost 10-fold higher than gonorrhoea, the relative increase in 2022 and 2023 compared with earlier years (2018–21) was notably lower than that of gonorrhoea, which sharply increased in 2022 (46%) and then slightly increased further in 2023 (7%) compared with 2022. Although the number of PCR tests performed increased from 2022, this does not account for the increase in cases. The number of gonorrhoea tests from 2018–23 is presented in Supplementary Figure S1 for comparison. The discrepancy in the relative incidence of the two sexually transmitted pathogens is striking, as this could indicate circulation in distinct reservoirs, or differences in selection mechanisms, varying manifestations and/or symptoms. The COVID-19 pandemic caused a significant reduction in overall infections in 2020 and subsequent lifting of restrictions from 2022 has been associated with an increase in not only respiratory infections [7,8] but also cases of gonorrhoea, as reported in England [9]. In Denmark, the final COVID-19 restrictions were lifted February 2022, which likely prompted behavioural changes that may have contributed to the sharp increase in gonorrhoea cases.

Data are extracted 9 January 2024 from the Danish Microbiology Database (MiBa), which contains copies of reports of analyses from all Danish departments of clinical microbiology.

## Geographical distribution and routes of transmission of *Neisseria gonorrhoeae*

Epidemiological data for 2022 showed several geographical hotspots, including the Capital region and Central and Northern regions of Jutland. The number of cases in 2022 by region of Denmark is presented in Supplementary Figure S2. In Northern Jutland, a 92% increase in gonorrhoea cases from 2021 (n = 279) to 2022 (n = 531) was observed, the largest increase among all five Danish regions. However, this sharp increase in cases nationwide did not continue in 2023 according to data extrapolated from the first 6 months, where only a minor increase was seen in Central Jutland and Zealand. No significant difference in age distribution in cases with gonorrhoea was observed from year to year (data not shown).

**Figure 2:**
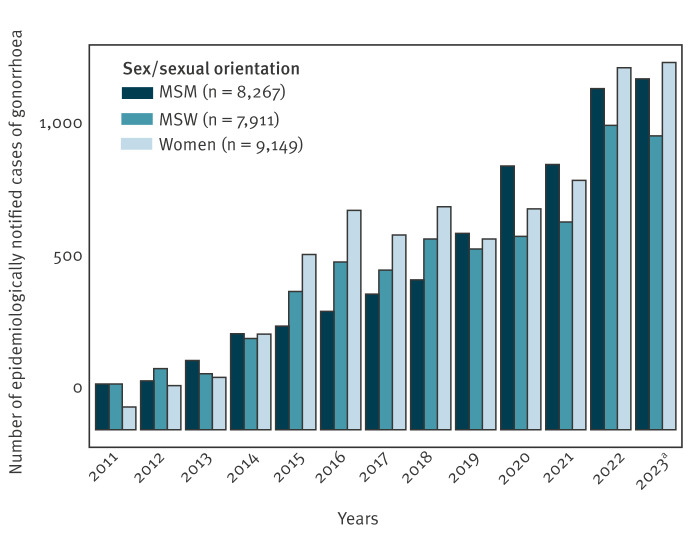
Notified gonorrhoea cases by sex/sexual orientation, Denmark, 2011–2023^a^ (n = 25,327)

MSM: men who have sex with men; MSW: men who have sex with women.

As reported from across Europe, including the Netherlands, Norway and Ireland [3], the increase in gonorrhoea cases in Denmark in 2022 was observed among both men and women, but a larger increase was seen among women and MSW compared with men who have sex with men (MSM) (Figure 2).

Epidemiological notified cases of gonorrhoea in Denmark and reported route of transmission.

^a^ In 2023, final case numbers were not available and thus extrapolated from the first 6 months of 2023.

## Molecular and epidemiological characterisation of *Neisseria gonorrhoeae*

To explore any underlying evolutionary determinants linked to the increase in gonorrhoea cases especially among women and heterosexual men, we selected 331 representative *N. gonorrhoeae* isolates, regardless of clinical sampling site, and performed whole genome sequencing. These isolates were all selected based on sampling date (2018–23), geographical region and availability of epidemiological data. There was an overrepresentation of Central and Northern Jutland regions for phylogenetic characterisation. This geographical selection, which was not based on the route of transmission, resulted in an obvious underrepresentation of MSM overall due to the larger prevalence of MSM in the Capital region. This should have no direct impact on the interpretation of the genomic results otherwise. For the remaining three regions, the number of included isolates for genome sequencing from each region was decided based on the total number of regional gonorrhoea cases over the study period, therefore every ca 30^th^ consecutive isolate from each remaining region was selected and genome-sequenced (Illumina, Inc.). The numbers of isolates sequenced from each region are presented in Supplementary Table S1.

**Figure 3:**
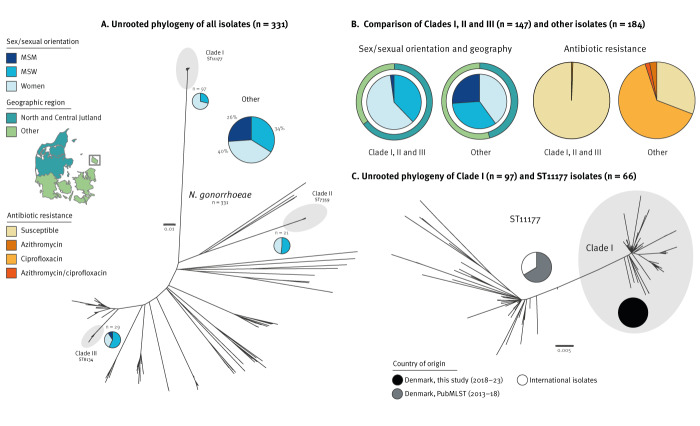
Population structure and epidemiological investigations of *Neisseria gonorrhoeae*, Denmark, 2018–2023

MSM: men who have sex with men; MSW: men who have sex with women; *N*: *Neisseria*.

All sequencing data were parsed through bifrost (https://github.com/ssi-dk/bifrost) for quality control, after which a core genome SNP-based phylogeny was obtained after purging for recombination. Overlaying of epidemiological data regarding sex, age and reported sexual orientation highlighted three distinct clades (I, II, and III, n = 147, Figure 3A) that all showed a significant overrepresentation of heterosexual transmission (99% MSW or women) compared with isolates outside these clades (76%). A comparison to general surveillance data in Denmark during 2018–23 strongly suggested that these ‘Other’ (n = 184) isolates were highly representative in terms of sexual orientation and phenotypic resistance to Danish cases overall in this period (Table; see Supplementary Figure S3 for a graphical representation). Sampling year and geographical distribution among the three clades are available in Supplementary Table S1. *Neisseria gonorrhoeae* circulating among MSM has been reported to exhibit higher levels of resistance [10], an observation that the Danish data support.

A. Unrooted phylogeny based on 28,752 SNPs across 89% of the reference chromosome conserved among all 331 *Neisseria gonorrhoeae* isolates sampled and genome-sequenced from across Denmark and linking with sexual orientation. Predominant sequence types of Clades I, II and III are highlighted.

B. Comparison of reported sex, sexual orientation, geography and phenotypic antibiotic resistance between Clades I, II and III and all remaining genome-sequenced isolates. Pairwise single nucleotide polymorphism (SNP) distances among the three clades were 0–79 (I), 0–70 (II) and 0–91 (III).

C. Unrooted phylogeny based on 1,290 SNPs between all Clade I isolates (n = 97) and all available ST11177 isolates (n = 66) from PubMLST. Clade I isolates are highlighted, as are the relative origin of isolates across the two observed clusters in the phylogeny. Scale bars indicate substitutions per site.

**Table:** Characteristics of the genome-sequenced *Neisseria gonorrhoeae* cases, Denmark, 2018–2023 (n = 331)

Characteristics	National surveillance 2018–23	Clade I (n = 97)	Clade II (n = 21)	Clade III (n = 29)	Other (n = 184)
Age in years, median (range)					
MSW	26 (13–82)	23 (17–47)	24 (15–64)	35 (18–69)	30 (17–69)
MSM	33 (16–80)	NA	NA	40 (30–50)	36 (18–63)
Women	23 (0–78)	23 (16–41)	23 (18–27)	26 (18–53)	26 (16–63)
Reported route of transmission (%)					
MSW	30.2	29	52	59	34
MSM	34.4	NA	NA	7	26
Women	35.4	71	48	35	40
Antibiotic resistance (%)					
Azithromycin	3.5	0.0	0.0	0.0	5.4
Ciprofloxacin	42.5	0.0	0.0	3.4	65.8
SNP distance					
Pairwise distance, min–max		0–79	0–70	0–91	0–3,930

MSM: men who have sex with men; MSW: men who have sex with women; NA: not applicable. SNP: single nucleotide polymorphism.

The median age of women, MSM and MSW and the relative proportion of the reported route of transmission within the individually defined clades are reported. The ‘National surveillance 2018–23’ data include all available Danish surveillance data representing resistance data from 18,118 isolates and sexual orientation reported from 17,225 cases between 2018 and 2023.

Single nucleotide polymorphism distance refers to the pairwise SNP distance between isolates based after purging of recombinant regions.

Unsurprisingly, the female population was younger than both the MSW and MSM populations across the collection, whereas when investigating the three clades, women and MSW were younger in Clades I and II compared with III (Table). Regarding antimicrobial susceptibility among all isolates in Clades I, II and III, only one isolate displayed phenotypic resistance. This was one of two isolates among the three clades collected from MSM. On the contrary, the remaining *N. gonorrhoeae* population exhibited extensive ciprofloxacin resistance (n = 121; 66%) but only sporadic azithromycin resistance (n = 10; 5%), of which only four (2%) were resistant also to ciprofloxacin (Figure 3B). No resistance towards ceftriaxone has been observed in Denmark since 2017. Recent recommendations in Denmark for the treatment of chlamydia include the use of doxycycline, but whereas genotypic analyses revealed the presence of *tet*(M) in 57 *N. gonorrhoeae* isolates, none of these clustered in Clades I, II or III.

Clade I was the most prevalent clone and consisted of isolates with sequence type (ST)11177 (85%; 82/97) and ST16478 (a single-locus variant of ST11177, 15%; 15/97). Isolates in this clade were observed throughout the 6-year study period in 18–42% (mean: 29%) of the sequenced isolates. The ST11177 has been reported in Ukraine [11], where it was the second most prevalent ST (26%) during 2013–18. Similar to the Danish isolates, all Ukrainian isolates of this type were reported as highly susceptible. However, the underlying genomic data from Ukraine are not publicly available for comparison.

The site PubMLST.org [12] contains 66 ST11177 *N. gonorrhoeae* genomes from Belarus, Denmark, Norway, Sweden, the United Kingdom (UK) and the Netherlands, suggesting that it has disseminated throughout large parts of Europe. A combined genome-based comparison highlighted that all Clade I isolates formed a monophyletic clade, with additional Danish isolates from previous years (2013–18) scattered throughout the rest of the phylogeny (Figure 3C). A representative ST11177 (Clade I) isolate (ID 632–2023) was subjected to long-read Nanopore sequencing (Oxford Nanopore Technologies) and a closed hybrid assembly genome has been made publicly available for future genomic investigations.

## Discussion

Our national data from Denmark show the evolving landscape of *N. gonorrhoeae* and demonstrate that the increase in gonorrhoea was not exclusively linked to MSM, but rather to MSW and women, and especially younger women in Clades I (ST11177) and II (ST7359). The unique combination of genomic and epidemiological data that include sexual orientation clearly depicts that distinct lineages have increased in prevalence in different geographical regions of Denmark and almost exclusively disconnected from the MSM community.

Despite the overall increase in gonorrhoea, the surveillance data on phenotypic resistance reveal that those lineages are highly susceptible, again in contrast to the variants circulating especially among the MSM community. Localised outbreaks have been reported [13], but with limited availability of genomic data, it is currently unclear if the lineages that drive the increase of gonorrhoea in MSW and women elsewhere in Europe all share common genetic backgrounds. The recent increase of gonorrhoea relative to chlamydia cannot be explained by antibiotic resistance among the key *N. gonorrhoeae* lineages. Rather, we hypothesise that these lineages may cause infection with no or low-grade symptoms and/or higher transmissibility. In addition, they may have become more prevalent in populations that are younger, more sexually active, and have multiple partners.

## Conclusion

Knowledge of distinct *N. gonorrhoeae* clones and their circulation in certain demographic groups aid in our understanding of transmission patterns, as does the observation of susceptibility of *N. gonorrhoeae* among these clades that can inform targeted interventions and identify populations at risk. Public health efforts may benefit from tailored strategies addressing specific populations, age groups, and geographical areas to mitigate the spread of gonorrhoea and optimise antimicrobial resistance management.

## Supplementary Data

146000 Supplement
