# Supplementary material for: Gonorrhoea on the rise in Denmark since 2022: distinct clones drive increase in heterosexual individuals
Source: Euro Surveill. 2024 Feb 15;29(7):2400059. doi: 10.2807/1560-7917.ES.2024.29.7.2400059 (PMC10986658; doi:10.2807/1560-7917.ES.2024.29.7.2400059)
Supplement: Supplement [file 24-00059_STEGGER_Supplement.pdf]

This supplementary material is hosted by Eurosurveillance as supporting information alongside the article ”Gonorrhoea on the rise in Denmark, since 2022: Distinct clones drive sharp increase in heterosexual individuals” on behalf of the authors who remain responsible for the accuracy and appropriateness of the content. The same standards for ethics, copyright, attributions and permissions as for the article apply. Eurosurveillance is not responsible for the maintenance of any links or email addresses provided therein.

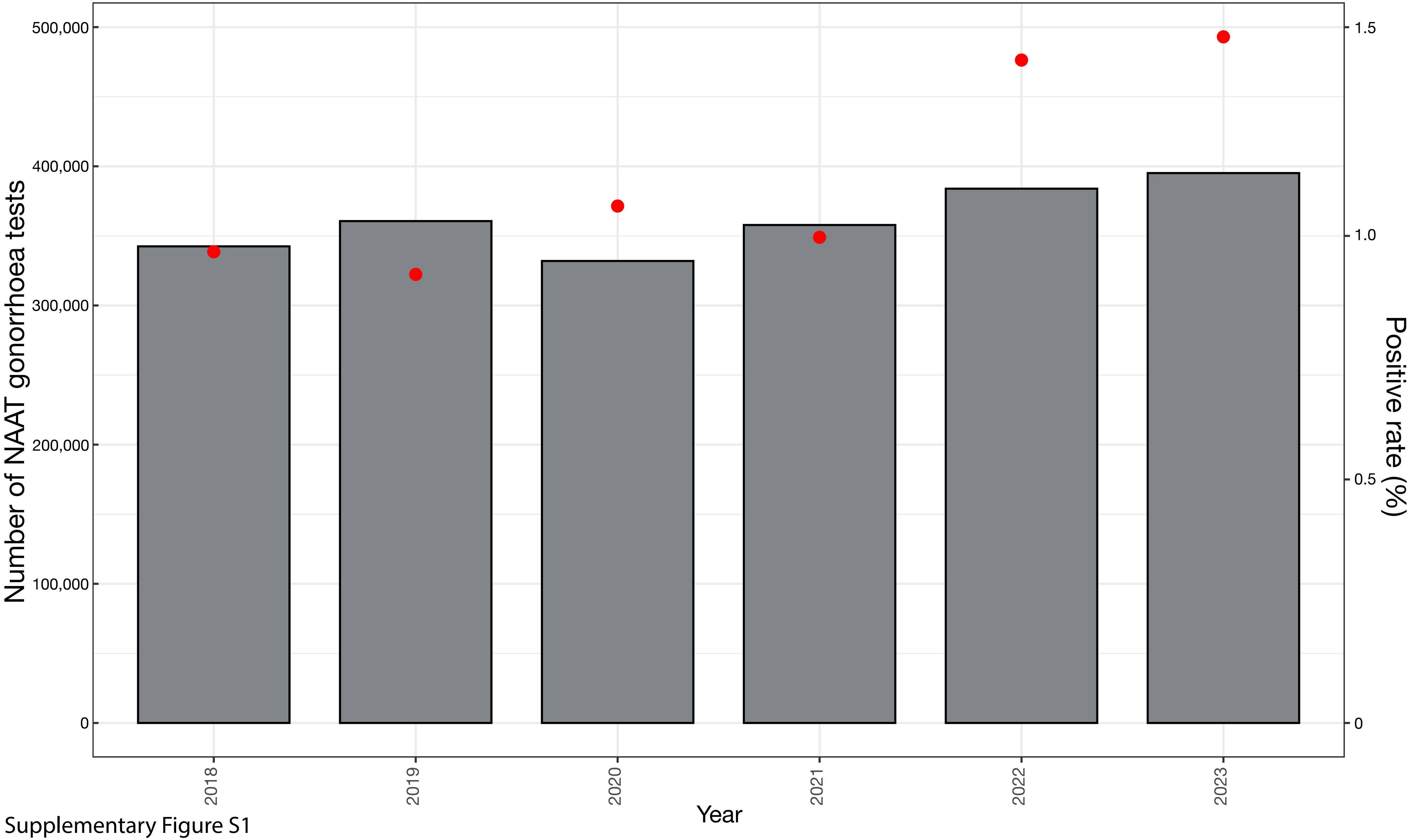

Supplementary Figure S1

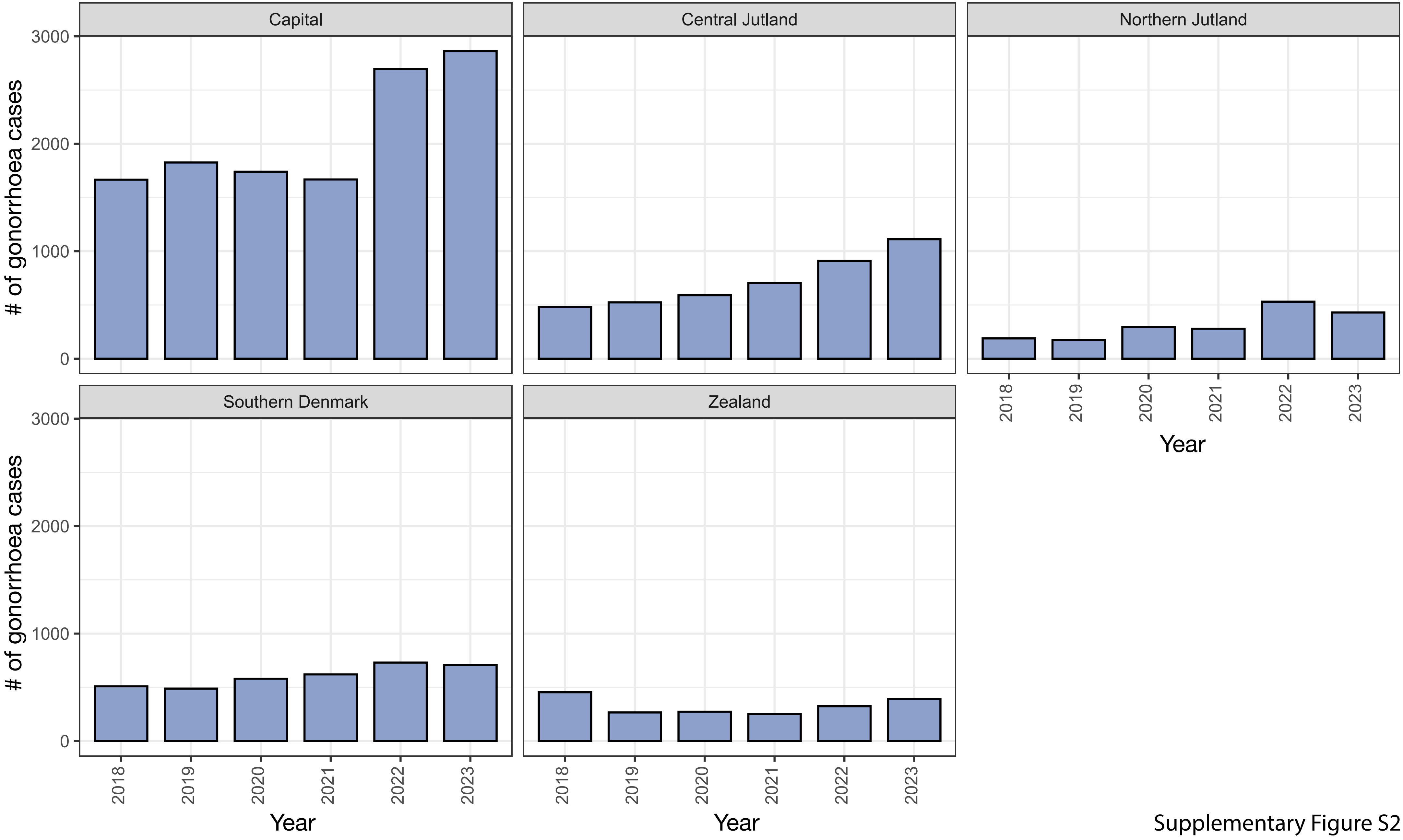

Supplementary Figure S2

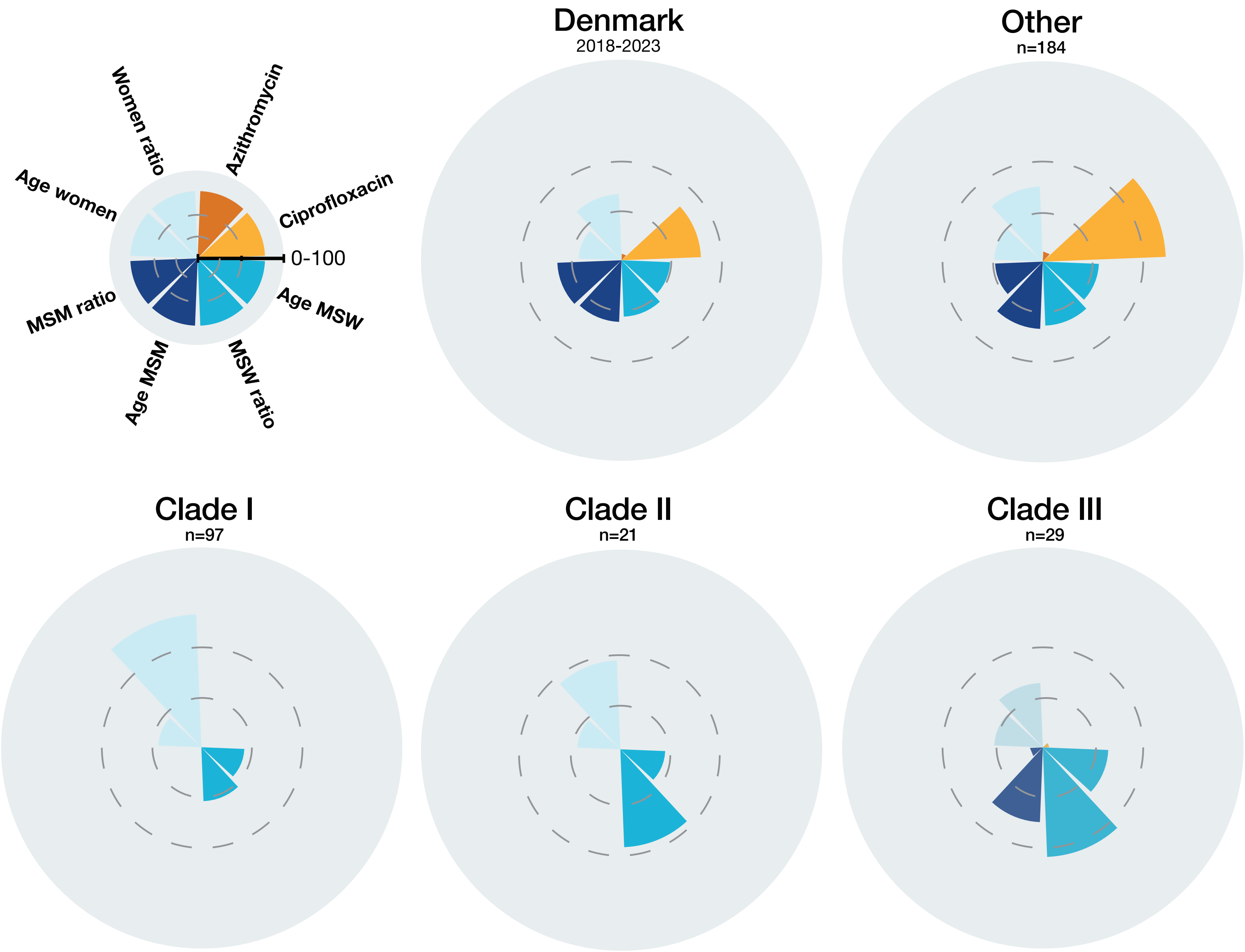

Supplementary Figure S3

	Clade I (n=97)	Clade II (n=21)	Clade III (n=29)	Other (n=184)	Total (n=331)
Sampling year					
2018	6	3	0	24	33
2019	10	0	0	22	32
2020	13	0	5	26	44
2021	23	3	0	29	55
2022	32	9	15	52	108
2023	13	6	9	31	59
Region					
Capital	13	3	8	67	91
Central Jutland	25	12	5	52	94
Northern Jutland	35	3	15	33	86
Zealand	5	1	1	8	15
Southern Denmark	19	2	0	24	45

Supplementary Table 1: Distribution of sampling year and region for Clade I, Clade II, Clade III, all “Other” isolates, and total.
